# 

**Published:** 1868-11

**Authors:** 


### Books and Pamphlets Received.

The Science and Practice of Medicine. By William Aitkin, M. D., Edinburgh, Professor of Pathology in the Army Medical School. Second American from the fifth, enlarged and carefully revised London edition, adopting the new nomenclature of the Royal College of Physicians of London, with large additions. By Meredith Clymer, M. D., Ex-Professor of the Institutes and Practice of Medicine in the University of New York, formerly Physician to the Philadelphia Hospital, etc., etc., in two volumes, with a map, lithographic plate and numerous illustrations on wood, Vol. II. Philadelphia: Lindsay & Blakiston, 1868. Received through, and for sale by Theodore Butler & Son, Buffalo.

The Medical Formulary, being a collection of Prescriptions derived from the writings and practice of many of the most Eminent Physicians in America and Europe—together with useful Dietetic Preparations and Antidotes for Poisons. To which is added an appendix on the Endemic use of Medicines, and on the use of Ether and Chloroform. By Benjamin Ellis, M. D., late Professor of Materia Medica and Pharmacy in the Philadelphia College of Pharmacy. Twelfth edition, carefully revised and improved. By Albert H. Smith. M. D., Fellow of the College of Physicians of Philadelphia, Lecturer on Obstetrics to the Philadelphia Lying in Charity, etc. Philadelphia: Henry C. Lea, 1868. Received through, and for sale by Theodore Butler & Son, Buffalo.

Outlines of Physiology, human and comparative, by John Marshall, F. R. S., Professor of Surgery in University College, London, Surgeon to the University College Hospital, with additions. By Francis G. Smith, M. D., Professor of Institutes of Medicine in the University of Pennsylvania. Illustrated with numerous wood cuts. Philadelphia: Henry C. Lea, 1868. Received through, and for sale by Theodore Butler & Son, Buffalo.

A treatise on the Principles and Practice of Medicine. Designed for the use of Practitioners and Students of Medicine. By Austin Flint, M. D., Professor of the Principles and Practice of Medicine in the Bellevue Hospital Medical College, Fellow of the New York Academy of Medicine, etc. Third edition, thoroughly revised. Philadelphia: Henry C. Lea, 1868. Received through, and for sale by Theodore Butler & Son, Buffalo.

A Collection of the Published Writings of the late Thomas Addison, M. D., Physician to Guy's Hospital. Edited with introductory prefaces to several of the papers, by Dr. Wilks and Dr. Baldy. The New Sydenham Society, London, 1868. Received through, and for sale by Theodore Butler & Son, Buffalo.

On Diseases of the Skin, including the Exanthemata. By Ferdinand Hebra, M. D., etc., etc. The New Sydenham Society, London, 1868. Received through, and for sale by Theodore Butler & Son, Buffalo.

A Treatise on Physiology and Hygiene, for Schools, Families and Colleges. By J. C. Dalton, M. D., Professor of Physiology in the College of Physicians and Surgeons, N. Y. With illustrations. New York: Harper & Brothers, Publishers, 1868. Received through, and for sale by Theo. Butler & Son, Buffalo.

The Opium Habit, with suggestions as to the remedy. New York: Harper & Brothers, Publishers, Franklin Square, 1868. Received through, and for sale by Theodore Butler & Son, Buffalo.

Retinitis Nyctalopica. By Prof. Dr. Arlt, of Vienna. Philadelphia: Lindsay & Blakiston, 1868.

The Materia Medica in its Scientific Relations. New Haven, Conn.: Judd & White, 240 Chapel Street, 1868.

Correlation of the Physical and Vital Forces. An Inaugural Address, introductory to the Course on Institutes of Medicine in the Jefferson Medical College, delivered October 12, 1868. By J. Aitken Meigs, M. D., Professor of the Institutes of Medicine and Medical Jurisprudence, one of the Physicians to the Pennsylvania Hospital, etc. Philadelphia: Office of the Medical and Surgical Reporter, 1868.

Doctor or Doctress? By Samuel Gregory, A. M., M. D., Secretary of the New England Female College. Boston: Published by the Trustees, and to be had at the College, 21 East Canton St., 1868.

Report of the Proceedings of the Association of Medical Superintendents of American Institutions for the Insane, at their Twenty-second Annual Meeting, held at Boston, Mass., on the 2d, 3d, 4th and 5th days of June, 1868. Harrisburg: Theo. F. Scheffer, Printer and Bookseller, 1868.

Brain Matter and Mind Action. Read before the St. Louis Medical Society, Oct. 10, 1868, by A. J. Steele, M. D., etc., etc.

The Transactions of the American Medical Association, vol. xix, 1868.

Late Publication—We must apologize for our Journal appearing a few days later than usual. Unavoidable delay, both in furnishing the copy and it publication when furnished, combine to make our excuse.

